# Barriers to men’s participation in perinatal care: a qualitative study in Iran

**DOI:** 10.1186/s12884-019-2201-2

**Published:** 2019-01-28

**Authors:** Vahideh Firouzan, Mahnaz Noroozi, Ziba Farajzadegan, Mojgan Mirghafourvand

**Affiliations:** 1 Faculty of Nursing and Midwifery, Isfahan University of Medical Sciences, Isfahan, Iran; 2 29 Bahman Hospital Research Center, Iranian Social Security Organization, Tabriz, Iran; 3 Department of Midwifery and Reproductive Health, School of Nursing and Midwifery, Isfahan University of Medical Sciences, Isfahan, Iran; 4 Department of Community Medicine, Medicine School, Isfahan University of Medical Sciences, Isfahan, Iran; 5 Social Determinants of Health Research Center, Tabriz University of Medical Sciences, Tabriz, Iran

**Keywords:** Men, Father, Participation, Perinatal care, Iran

## Abstract

**Background:**

The role of men’s participation in prenatal, delivery and postpartum care is very important, well defined and cannot be over emphasized. Very few studies exist about men’s role in promoting the health of the mother and barriers to their participation in perinatal care in Iran; hence, the present study was conducted to determine the barriers to men’s participation in perinatal care.

**Methods:**

The present qualitative study was carried out on 45 participants who were selected employing purposeful sampling technique. Data were collected through in-depth semi-structured interviews, focused group discussions and field notes. Data were analyzed using conventional content analysis.

**Results:**

After data analysis, four main categories extracted were: “cultural barriers”, “personal and interpersonal barriers”, “health system-related barriers” and “socio-economic barriers”.

**Conclusion:**

The results of this study, by presenting the barriers to and challenges for men’s participation in perinatal care, could be helpful in designing culture-based strategies to overcome these barriers and improve men’s participation.

## Background

In women’s life, reproductive period which include preconception, pregnancy, delivery and postpartum period, is a critical period; because, despite positive expectations for its outcomes, it might lead to disability or even death [1]. Men’s participation during the perinatal period is of great importance in the health of the mother and the infant. According to previous studies, men’s participation would decrease the rate of preterm birth, low birth weight (LBW), restricted fetal growth, neonatal mortality and mother’s stress and increase the rate of prenatal and postpartum care [2]. Furthermore, men’s participation in services related to the health of the mother and the infant would lead to early performance and completion of the prenatal care, improvement of mother’s workload during pregnancy, preparation for delivery and improvement of the couple’s relationship [3–5]. Men’s participation in the healthcare services for mothers is a social and behavioral course of action that should be performed actively and responsibly to improve the health of the mother and the infant [6].

In most parts of the world, men are responsible for making important decisions regarding the allocation of financial resources and care behaviors that could directly affect the health of the mothers and the infants [7]. Also, men’s behaviors would affect the reproductive health of their wife and children. However, most of the maternal and child health programs were focused on women’s education and participation while men were neglected [4]. Although in the International Conference on Population and Development (ICPD) in Egypt, 1994, and the Fourth World Conference on Women, the positive role of men in the health and reproductive rights of men was emphasized [8] and the importance of men’s participation in maternal and child health services was approved by most of the countries; the rate of this participation was very low [4] and there were barriers to accomplishing this matter. According to the conducted studies, some of the recognized barriers were educational level, income, health center-related factors and low awareness about the role of men in reproductive health [2, 5, 9, 10]. Furthermore, factors such as perceptions, beliefs and attitudes toward maternal health as a feminine duty would lead to poor participation of men in reproductive health [9–11]. Most countries have encountered challenges at executive levels in this field and more studies are required to determine the barriers and effective interventions for resolving them for improved men’s participation. Also, in Iran, the level of men’s participation in related issues to the maternal and fetal health is low and few studies were conducted regarding the causes for low participation of men and barriers to their participation. Thus, performing more studies in this field was necessary for designing culture sensitive and effective interventions on men’s participation during pregnancy, delivery and postpartum period. This could help to improve the health of the mother and the infant.

Qualitative research is an approach for discovering and describing people’s experiences and conceptualizing them, such that would increase the imminent understanding and awareness about human experiences. It is usually used when there is a need for explaining concepts and the relation between them [12]. Thus, the present qualitative study was conducted to determine the barriers to men’s participation in perinatal care.

## Methods

The present study is a qualitative research that was conducted employing content analysis approach between July and November 2017.

### Settings, sample and recruitment

In this study participants include pregnant women or women had just delivered (*n* = 12), husbands of pregnant women or women who had recently experienced delivery (*n* = 6), healthcare providers include midwives, gynecologists and nurses (*n* = 19) in Tabriz, Iran, deputy health managers at Tabriz University of Medical Sciences (*n* = 3) and policymakers from the Ministry of Health and Medical Education (*n* = 5). The inclusion criteria were having at least five years of working experience with caregivers, being able to understand and express their experiences and having Iranian nationality. Eligible Participants (pregnant women or women had just delivered, husbands of pregnant women or women who had recently experienced delivery, and healthcare providers) were accessed through postpartum wards of hospitals, prenatal clinics and midwife or gynecologist offices. They were recruited directly or telephone numbers were obtained and they were called. The sampling started using purposeful sampling technique and continued considering the maximum variety in educational level, socioeconomic condition, job, age, and number of pregnancies and deliveries (for pregnant women or women had just delivered). Also, healthcare providers as well as deputy health managers and health policy makers with different working experience and from different health centers recruited for the study. The selected participants were willing to participate in the study and informed consent was obtained from each them. The demographic characteristics of the 45 participants are shown in Table 1.

**Table 1:** Participants’ demographic characteristics

Age 15–55	
Gender Female (36), Male (9)	
Educational level Associate’s degree and Bachelor’s degree (17) Middle school degree (1), Diploma (5) Master’s degree and Ph.D (22)	
Occupational status Employee (35), Housewife (8), Freelancer (2)	
Number of children 0–3	
Status of the Delivered (28), Pregnant (13) participant or their spouse With a history of pregnancy or delivery (1) Without a history of pregnancy or delivery (3)	

### Data collection

Data were gathered through face-to-face in-depth semi-structured interviews, focused group discussions (FGDs) and field notes. First author (VF) conducted the interviews and focused group discussions. She had 20 years working experiences with pregnant women and she had taught in “preparation for labor and childbirth” classes in Tabriz city since 10 years ago. Other authors had previous interviewing experience and qualitative paper/report writing. Prior to data collection, first author wrote down initial preconceptions and beliefs about research topic based on their previous working experiences with pregnant mother and from a review of the literature. These preliminary thoughts and ideas led to the providing an interview guide.

Interviews began with the open question of “What are the men’s participation barriers in antenatal and postpartum care? Please explain.” Then, the course of the interviews was guided by the participants’ open and interpretative answers. In addition, as the interviews went on, more detailed questions (based on the interview guide) were asked. The places and time for the interviews were selected based on the participants’ preferences. The interviews lasted between 25 to 100 min and continued until there was data saturation and no new data code emerged in the interviews and FGDs. Two FGDs were conducted. One of them was conducted for eight pregnant women and lasted 105 min and another was conducted for seven health providers and lasted 120 min (Table 2). The time and place of both group discussions were determined by the opinions of the participants. At the group discussions sessions, the researcher, was acting as the facilitator and guider of the discussions, and another person was present to take notes.

**Table 2:** Participants’ characteristics in FGDs

Characteristic	Age (years)	Educational level	Occupational status	Number of pregnancy	Trimester of pregnancy	Working experience (years)
Pregnant women (*n* = 8)	18–38	Middle school degree (1) Diploma (3) Associate’s and Bachelor’s degree (4)	Housewife (5) Teacher (2) Nurse (1)	First pregnancy(6) Second pregnancy (2)	First trimester (1) Second trimester (5) Third trimester (2)	–
Health providers (*n* = 7)	38–55	Bachelor’s degree (2) Master’s degree (5)	Midwife (7)	–	–	12–27

### Data analysis

Data analysis was performed simultaneously with data collection. All interviews were digitally recorded. The interviews were transcribed verbatim by first author (VF). The data obtained were analyzed employing conventional qualitative content analysis [13]. This technique is appropriate for subjective interpretation of the data text content through regular process of coding to determine the categories or themes. In this regard, interviews were repeatedly reviewed to achieve a comprehensive approach for the data. The transcriptions of the interviews were divided into meaning units, and after compression; they were labeled with abstract codes. Codes were compared with each other regarding their similarities and differences and were then classified into sub-categories and categories. In this study, in several interviews, the coding were separately performed by the first and second author, and then continued by both authors agreement. In all cases, other authors, after analyzing the data by the first and second authors, commented on codes, subcategories, and categories.

### Rigor and trustworthiness

To ensure the credibility to the results, the data was validated by establishing a close relationship with the participants as well as gaining their trust. Also, sampling with maximum variation increased the validity of the data. As member checking, after formation of the primary codes, the opinions of four participants were sought to approve the accuracy of the codes and interpretations. To ensure the transferability of the data, opinions of three individuals who had similar characteristics to the participants but were not enrolled in the study, were sought. Dependability of the data was approved after review by the research team members. In addition, the data were reviewed by four experts in this field who were not involved in this study; hence, the accuracy of the coding process was evaluated.

### Ethical considerations

Ethical approval of the research was received from the Ethics Committee of Isfahan University of Medical Sciences (ethical approval code: IR.MUI.Rec.1395.3.599). The reasons for the study were explained prior to each individual interview and FGDs. Informed consent, anonymity, confidentiality and the right of participants to leave the study at any time was preserved.

## Results

During data analysis, 247 codes and 10 sub-categories developed that were eventually divided into four main categories of “cultural barriers”, “personal and interpersonal barriers”, “health system-related barriers” and “socio-economic barriers” were analyzed (Table 3).

**Table 3:** Results of data analysis

Codes	Sub-category	Category
• Negative attitudes toward the issue of male participation • Men’s fear of social stigma • The community believe of the responsibility of the woman	The undesirable dominant socio-cultural climate of the society	Cultural barriers
• Unwillingness to allow husband’s presence in maternity ward and prenatal clinic • The traditional women’s reliance on their family	Lack of request from women	
• The insignificant contribution of families in learning to participate in boys • The lack of education of men and boys in our society	Educational poverty in the society	
• Unawareness of men about the importance of their collaborative role in the perinatal period • Men’s insufficient experience about participation in pregnancy, childbirth and postpartum care • The lack of men’s preparation at early age for fatherhood	Poverty of consciousness and men’s inadequate experiences	Personal and Interpersonal barriers
• Changing the sexual function of couples during pregnancy • The lack of proper interaction between couples	Couple’s communicational problems	
• Lack of appropriate physical space in health centers for men • Inappropriate time to provide prenatal care for the possibility of male participation • Restriction and even the ban on men for entering the prenatal clinic	The structural problems in the health centers	Health system-related barriers
• The negative attitude of the health providers towards the presence of men alongside the wives in health centers • The lack of male reproductive health professionals for the education of boys and men • The embarrassment of female medical staff to confront men in women’s care settings	Problems related to the human resources	
• Applying the opinion and personal preferences of the senior managers of health centers to avoid the presence of men in these centers • The lack of attention of policymakers to the issue of men’s participation in perinatal care in large-scale planning and policy as a community’s need despite its acceptance • The lack of a specific program in the health ministry’s plans for male participation in perinatal care	Policymaking and managerial problems	
• Lack of time for men due to long working hours • Failure to grant and perform paternal leave	Men’s occupational problems	Socio-economic barriers
• High costs of pregnancy care and childbirth • The lack of financial support for families during pregnancy by the government • Low economic level of the family	Financial problems	

### Cultural barriers

Based on the participants^,^ narrations, cultural barriers were the most important and common reason for men’s nonparticipation in perinatal care. This main category was made up of three sub-categories.

### The undesirable dominant socio-cultural climate of the society

Most of the participants mentioned the existence of gender roles in the society and the traditional patriarchy culture which is dominant in the Iranian families; they also mentioned the negative attitude toward men’s participation in pregnancy, delivery and postpartum care. Participants mentioned men’s fear of the social stigma for participating in pregnancy and delivery care, as a barrier to their participation during this period. Participants also mentioned society’s and even women’s belief about women’s responsibility in care and household matters. However some of the participants stated more participation among young men compared to others.

*“Women in the families, especially husband’s family, believe that delivery is a woman’s duty; having a baby has nothing to do with the husband at all; woman should take care of the child, she is responsible for the child. So this belief would unconsciously be induced in the husband that he does not need to help, he should just provide the finances while senior women of the family would take care of the caring matters.” (Pregnant woman).*

### Lack of request from women

Male participants mentioned that one of the main reasons for lack of men’s participation in pregnancy and delivery matters was lack of request from women and that women would rather rely on their own families. Some of the pregnant women who participated also mentioned women’s unwillingness for the presence of their husband at the delivery room and prenatal clinics, to maintain their privacy.

“*I did not want my husband to be by my side during labor pains because you would not act normal during labor and might do irrational things. I remember that I was crawling out of pain or screaming for example; that’s why I did not want my husband to see me like that.” (Gynecologist).*

### Educational poverty in the society

According to most of the participants, families have a small role in teaching their sons about participation; instead, they usually teach men not to participate in pregnancy and delivery matters. They believed that the media has a weak role in creating the culture of men’s participation in prenatal care (for considering the pregnancy and delivery as private topics). The women who participated also believed that the timing for airing the few shows in this field was inappropriate; meaning that only women could be watching these programs. Also the mass media has mostly portrayed the caring role of the mother and the authoritative role of the father, while the fatherly role of men was often neglected.

*“… for example the only scene that would be shown from a woman’s pregnancy in a TV show is her nausea; then she would be brought to the operating room and boom, that’s the delivery. So, little boys would not see anything about men’s participation in the movies and TV while growing up that would teach them.” (Policymaker).*

### Personal and interpersonal barriers

Based on the participants^,^ narrations, lack of men’s awareness about the importance of their participation in perinatal period, and their role in mother and infant health, lack of adequate experience about participation in pregnancy, childbirth and postpartum care and lack of proper interaction between couples due to lack of appropriate communication skills in life were another reasons for men’s nonparticipation in this regard. This main category was comprised of two sub-categories.

### Poverty of consciousness and men’s inadequate experiences

According to the participants, men’s unawareness about the importance of their role in prenatal period and their inability to understand the problems of pregnant women, would lead to their non-performance of their positive role during this period. Participants also believed that, men’s inadequate experiences for participating in pregnancy, delivery and postpartum period (especially during the first pregnancy) due to their young age or not being prepared for fatherhood were some other reasons for lack of men’s participation.

“*Men think that pregnancy is a repetitious and an ordinary fact; so, they would not care about it because they are not aware of pregnancy’s risks.” (Midwife).*

### Couple’s communicational problems

According to the participants, lack of appropriate communication between couples and the changes in their sexual performance during pregnancy would lead to communicational problems during pregnancy which would become a barrier to their participation during pregnancy and delivery period.

*“The main problem is in the couple’s communication skills, they should learn the manners of communications and talking about their needs and responding to their needs before marriage.” (Midwife).*

### Health system-related barriers

Based on the participants^,^ narrations, the barriers related to health system were the main hindrance for interested men’s participation in perinatal care. This main category was made up of three sub-categories.

### The structural problems in the health centers

Participants stated that the inappropriate physical environment of health centers for men’s presence. as well as the inappropriate time of providing pregnancy care for men’s participation, as the main reasons for not accompanying their wives during pregnancy, delivery and postpartum care. They mentioned the existence of restrictions or even prohibition for men’s presence at the examination rooms in prenatal clinics. Also, in the private offices of the gynecologists and midwives, husbands were not allowed to enter the examination room; because mothers would be visited in groups and maintaining the privacy of mothers would become a challenge. Furthermore, because the space of labor rooms is not private in most of the public hospitals, it is not possible for the husbands to be present. According to the participants, services at prenatal clinics would be provided during the morning shifts and men should be at work during these hours, therefore, the possibility of accompanying the wife during pregnancy care would be decreased.

“*The conditions are not prepared… If someone has the financial resources, they might be able to get private rooms in private clinics and be by their wife’s side, but in public centers, traditionally, legally and even structurally, husbands could not be by their wives during labor.” (Midwife).*

### Problems related to the human resources

Based on the participants’ statements, disapproval of men’s presence by healthcare providers during pregnancy care; labor and postpartum examinations and the gender of the healthcare providers were some of the reasons that would decrease men’s participation. Participants believed that the reasons for disapproval of men’s presence by the healthcare providers in the feminine environment of prenatal clinics and labor rooms were due to disruption of health care providers’ services, crowdedness of the clinics and shortage in healthcare staff for allocating sufficient time to men’s education as well as religious attitudes toward confrontation of female healthcare staff with men.

*“Midwives and physicians do not like men to enter the clinic or the labor room, because they would intervene with their work or they would ask questions that the physicians and the midwives could not answer them.” (Deputy Director of Treatment).*

### Policymaking and managerial problems

Participants mentioned that the neglect of men’s participation in prenatal care by policymakers in planning and macro policies, despite the need of the society; the application of the personal opinions and interests of the senior administrators of health centers to avoid men’s presence at these centers, were some of the barriers to men’s participation in pregnancy, delivery and postpartum care.

“*The policymakers and decision makers do not even think about men’s participation in prenatal cares; they see it as a need but they have never thought about planning or making policies for it, thus, they have not considered it in their policies.” (Policymaker).*

### Socio-economic barriers

Participants believed that men’s occupational problems and conflicts, as well as high living costs and family financial problems, make men less likely to be involved in perinatal care as they spend a lot of time outdoors. This main category was made up of two sub-categories.

### Men’s occupational problems

According to the participants, lack of sufficient time and strength due to the exhaustion of men, caused by long hours of work and the inapplicable plan of leave for fathers in most of the administrative organizations and institutes, were some of the barriers to men’s participation in pregnancy, delivery and postpartum care. Men participants believed that, even in the few organizations where this plan is superficially applied, lack of support of the employers for men who would want to use this leave and men’s fear of disturbing their job position if using the leave, would make them refuse to take the leave and consequently, men would be deprived from participating in the sensitive days following delivery.

*“Unfortunately, due to the financial problems, men have to work for long hours, so they are tired and exhausted when they come home; I myself would go home after working the morning and the evening, I am so exhausted I cannot even stand on my feet, let alone be helpful.” (Father).*

### Financial problems

Most of the participants stated that poor economic status of the families, high costs of pregnancy and delivery and not providing financial supports for the families during pregnancy by the government, were the reasons for men’s need to work multiple shifts or even multiple jobs; this would consequently decrease their participation during this period.

*“I work from 7 am to 8 pm. But if I would be financially secure, I would come home at 4 pm and help my wife. But due to the high costs of ultrasounds and pregnancy examinations, I would even appreciate working on Fridays.” (Father).*

## Discussion

The present study was conducted to determine the barriers to men’s participation in perinatal care. Results of the present study showed that the most important barriers to men’s participation in perinatal care were cultural barriers, personal and interpersonal barriers, health system-related barriers and socio-economic barriers. In Iran, like most other countries, cultural barrier was one of the important and fundamental barriers to men’s participation in perinatal care [4, 14–16]. Participants mentioned gender roles, negative attitude toward men’s participation, taboos and stigmas related to this issue in the society as the cultural barriers to men’s participation in perinatal care. It should be noted that in the traditional context of Tabriz in Iran, gender roles are defined in a way that create the dividing line between feminine and masculine affairs. Women are responsible for home affairs and the care of the children and men for earning income for the management of life. Additionally, senior women (such as mother- in –law) in the families of Tabriz are considered as the main agents in care and guardians of women during pregnancy, childbirth and postpartum care. The acceptance of these cases by men causes that they do not react to barriers to their participation in perinatal care and do not attempt to eliminate it. Although today’s men are more involved with perinatal care than in the past, they are still far from desirable.

Results of the studies by Mortazavi & Mirzaei [17] and Simbar et al. [18] showed that cultural barriers as the main hindrance to men’s participation in perinatal care; which was similar to the results of the present study. Also a study in Cameron showed that tribal beliefs and traditional gender roles were the barriers to men’s participation in prenatal care [16]. In a report that was published by the World Health Organization (WHO) in 2015 about the condition of the fathers in the world, social and cultural norms that would determine the roles of men and women was mentioned as a barrier to men’s participation in maternal and neonatal health [19–23]. Although some of the participants in the present study mentioned more participation of younger men than others in family matters, similar to a study that was conducted in Chile, the necessity of institutionalizing this participation during pregnancy, delivery and postpartum period in the family and the society was also mentioned by the participants [24]. It seems that the type of family trainings for boys that promotes the authoritative role of men in life, as well as lack of education for men about their participation in the society, especially through the mass media, were important factors in this field; it is probable that by emphasizing on the positive role of men in perinatal care in the society through the mass media, religious and social leaders, their participation would be improved. In this regard, in Malawi, husbands who were listening to radio programs about this issue had more participation in pregnancy, delivery and postpartum care than those who did not listen to the radio programs [25].

Personal and interpersonal barriers were another barrier to men’s participation in perinatal care; which was stated by participants as poverty in awareness, inadequate experience in men especially during the first pregnancy and communicational problems between the couples. A study showed that little knowledge of men and their lack of believe in the risks of pregnancy, were the barriers to their participation in prenatal care [21]. Also, A study in Myanmar showed that the existence of a positive correlation between men’s participation and their level of knowledge about reproductive health [26]. Other researchers in their study showed that despite men might not have knowledge and awareness about pregnancy and delivery; they could control the situation by making the right decisions at the time of emergency [27]. The results of studies also showed personal factors, family and social relationships as the barriers to men’s participation [2, 3]. This might be due to lack of communication skills between the couples, replacing effective communication between family members with social networking technology, and lack of mutual understanding of each other’s needs between the couples. On the other hand, traditional beliefs and concerns about risking the health of the mother and the fetus by having sexual intercourse during pregnancy and also wrong information provided by the healthcare staff, might lead to restraining sexual activities during pregnancy and after delivery; this might cause a distance between the couple which could lead to lack of participation by the husbands. It seems that conducting a consulting session at the beginning of the prenatal care and educating couples on the correct ways of communication and having sexual intercourse during pregnancy could increase men’s participation by eliminating these barriers.

Another barrier to men’s participation in perinatal care that was emphasized by the participants was health system-related barriers. Similar to another studies [4, 15, 19, 24, 28–30], in the present study structural problems of the health centers and the negative attitude of the female service providers toward men’s participation in prenatal, delivery and postpartum care were the major setbacks. Also, lack of couple-friendly structures in centers for providing prenatal and labor services was one of the barriers by the health system to men’s participation in perinatal care in the present study. A study showed that limitations of the health centers including limitations in personnel, space and attitude of the healthcare personnel toward men’s participation were some of the barriers to men’s participation in perinatal care [15]. Mortazavi & Mirzaei in their study stated that inappropriate behavior of the healthcare personnel was a barrier to men’s participation in perinatal care [17]. Kaye et al. in Uganda considered the health system intimidating, unwelcoming and unsupportive and mentioned the health system factors as the essential factors for promoting men’s participation in women’s health issues during pregnancy and delivery [2]. Therefore, it seems that existence of such barriers would even dissuade men who were intending to have an active role in their wife’s care and to be present in the hospitals and clinics. Thus, changing the attitude of the healthcare personnel, welcoming the presence of men in the health centers and educating communicational skills to the healthcare personnel to make appropriate communication with husbands, could be the solutions to increase men’s participation in perinatal care. Also, the modification of the physical structure of the clinics, such that patients could be examined privately along with their husbands, as well as modifying the structure of the labor wards, such that by maintaining the privacy of the mothers, husbands could be allowed in the labor ward too, could be helpful.

Regarding the policymaking and managerial problems, not officially recognizing the role of men in maternal and neonatal health, not paying attention to men’s participation in perinatal care in the macro policies and programming of the Ministry of Health Medical Education and also not correctly performing the existing policies in this field were some of the barriers that were mentioned by the participants. A study showed that although many husbands, particularly in urban areas, go to the clinic with their wives, they are rarely included in any consultations [4, 31]; Probably, signifying the role of fathers in the maternal and neonatal health and informing the public about their rights in this field could make this a public request and cause policy makers to think about designing a protocol in this field with focus on the role of fathers in maternal and neonatal health.

Socio-economic barriers were another barrier mentioned by the participants. All of the participants mentioned men’s occupational problems and lack of sufficient time for accompanying the wife during pregnancy, delivery and postpartum period, family’s financial problems, high costs of pregnancy and delivery cares and not providing any financial support from the government for the families who are expecting or have children as barriers to men’s participation in perinatal care. In a study men’s occupational problem was mentioned as one of the barriers to their participation [17]. It seems that decreasing the costs of pregnancy and delivery cares and para-clinical measures during pregnancy and financial supports from the government for expecting families would cause more relief for men and provide an opportunity for their participation during perinatal care. Participants in the present study emphasized on not correctly applying the law of two-week leave for newly fathers in most of the offices and organizations. It seemed that supervising the appropriate implementation of the two-week leave law for newly fathers and considering a motivational system for encouraging the managers who appropriately implement the law and also fathers who use the two-week leave, could overcome this barrier.

Generalization of the findings in the present study, considering its qualitative approach, should be done cautiously. Although qualitative studies are not made for generalization of the results, they might be important for those who are willing to use the results of these studies and could be considered as a limitation. Therefore, through selection of participants with maximum variations, guidance and supervision of experts and external reviewing, the effort was to increase the accuracy and transferability of the data.

## Conclusions

The present study revealed cultural, personal and interpersonal, socio-economic and health system-related barriers to men’s participation in perinatal care. By determining the barriers and emphasizing on them, probably, serious effort could be made for eliminating them. And by providing the results to policymakers and senior country managers, effective strategies for increasing men’s participation might be designed and consequently, maternal and neonatal health would be improved.

## Abbreviations

FGDs: Focused Group Discussions
ICPD: Conference on Population and Development
LBW: Low Birth Weight
WHO: World Health Organization.
